# Random Volumetric MRI Trajectories via Genetic Algorithms

**DOI:** 10.1155/2008/297089

**Published:** 2008-07-01

**Authors:** Andrew Thomas Curtis, Christopher Kumar Anand

**Affiliations:** ^1^Department of Medical Biophysics, The University of Western Ontario, London, ON, Canada N6A 5C1; ^2^Department of Computing and Software, McMaster University, Hamilton, ON, Canada L8S 4K1

## Abstract

A pseudorandom, velocity-insensitive, volumetric *k*-space sampling trajectory is designed for use with balanced steady-state magnetic resonance imaging. Individual arcs are designed independently and do not fit together in the way that multishot spiral, radial or echo-planar trajectories do. Previously, it was shown that second-order cone optimization problems can be defined for each arc independent of the others, that nulling of zeroth and higher moments can be encoded as constraints, and that individual arcs can be optimized in seconds. For use in steady-state imaging, sampling duty cycles are predicted to exceed 95 percent. Using such pseudorandom trajectories, aliasing caused by under-sampling manifests itself as incoherent noise. In this paper, a genetic algorithm (GA) is formulated and numerically evaluated. A large set of arcs is designed using previous methods, and the GA choses particular fit subsets of a given size, corresponding to a desired acquisition time. Numerical simulations of 1 second acquisitions show good detail and acceptable noise for large-volume imaging with 32 coils.

## 1. INTRODUCTION

Reconstruction of magnetic resonance imaging (MRI) from data sampled using noncartesian sampling has recently received increasingly mathematically sophisticated treatment, for example [1], with notable improvements in reconstruction speed and accuracy. The application of novel design techniques for noncartesian sampling trajectories, however, has received less attention.

In [2], a novel pseudorandom volumetric *k*-space trajectory design method was presented. This methodology, henceforth referred to as Durga, combines a number of ideas in trajectory design and general sampling design for the first time, including randomness, [3, 4] constrained optimization [5] to balance trajectories for steady-state imaging [6–9], genetic algorithms [10], under-sampling to trade acquisition time for (structured) noise [11–13], and target-oriented design rather than patterns of symmetric interleaving [14, 15]. By combining these ideas, Durga achieves significantly better efficiency as measured by sampling duty-cycle for a balanced steady-state pulse sequence.

Flow-insensitive *k*-space trajectories are inherently more efficient than trajectories requiring a rewinder to balance first and possibly higher moments. Trajectories which sample three dimensions in *k*-space, like Durga, can further increase efficiency by not rewinding slice select gradients, and simply starting and stopping sampling offset from the center of *k*-space immediately after and before the excitation pulse. This is summarized in Table 1 by a comparison of spiral with velocity compensating rewinder [5], Teardrop [7] which incorporates in-plane velocity compensation into the readout, and Durga [2]. These numbers are relative to gradient peak/slew limits of 40 mTm^−1^ and 150 Tm^−1^s^−1^ for Spiral [5] and Durga [2] and 27 mTm^−1^ and 72 Tm^−1^s^−1^ for Teardrop [7].

Volumetric imaging has several inherent advantages over thin-slice imaging, including isotropic resolution, reduced effect of in-flow, and the ability to completely correct for distortions in gradients, main-field inhomogeneity, and eddy currents. It is also easier to completely compensate for velocity effects by nulling higher moments. Moment-nulled planar trajectories are often only effectively nulled in one or two dimensions, because they are used with slice select and possibly phase encoding gradients which are difficult to null.

In the case of rapid imaging, however, the most important property of sampling in more dimensions is the ability to under sample without introducing aliasing in the form of ghosts [11, 16]. Using Durga, one can essentially trade off under sampling for unstructured noise on a continuous basis, breaking the dependence on Nyquist sampling. The trajectories used in the illustrations to reconstruct 256^3^ volumes are 33× under sampled in time relative to perfect Cartesian sampling (ignoring gradient limits, and only considering sampling bandwidth).

This paper presents two innovations over [2].

**Figure 1:**
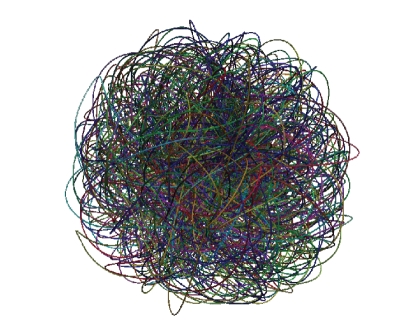
Tubes depicting the 178 selected trajectories.

1. A genetic algorithm for choosing subsets of trajectory arcs (Figure 1) corresponding to a *T*_*R*_, designed using one of the methods in [2]. This significantly increases the simplicity and flexibility of implementation and improves the quality of the solution, as measured by the point spread function.
2. Multicoil numerical simulations, including the effect of noise, showing low levels of aliasing artifacts with extreme under-sampling.

Although this sampling strategy is compatible with and benefits from multicoil imaging, the reduction in aliasing possible by using an iterative SENSE reconstruction [17] is much smaller than what could be expected based on experience with other trajectories.

## 2. METHODS

### 2.1. Individual trajectory design

**Figure 2:**
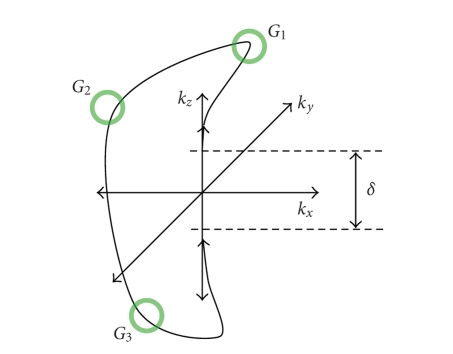
Diagram showing a trajectory going through specified goals at maximum resolution. *δ* is a gap in the sampling, chosen to correspond with the rf excitation.

Sampling trajectories in 3D *k*-space are generated by solving a second-order cone optimization problem (SOCP), originally formulated in [2]. An SOCP is a minimization problem in which the variables are constrained to lie inside a set defined by quadratic functions of the variables. Putting a bound on the length of a vector is a common special case, and the one exploited in this trajectory design problem:

(1) $$\begin{array}{ll} \min\; & \tau , \end{array}$$

(2) $$\begin{array}{ll} & k_{0}=0=k_{N}, \end{array}$$

(3) $$\begin{array}{ll} & ∥k_{i+1}-k_{i}{∥}_{2}\leq G_{\text{max}}\delta_{T}, \end{array}$$

(4) $$\begin{array}{ll} & ∥k_{i+2}-2k_{i+1}+k_{i}{∥}_{2}\leq S_{\text{max}}\delta_{T}^{2}, \end{array}$$

(5) $$\begin{array}{ll} & ∥k_{i}{∥}_{2}\leq 1/R, \end{array}$$

(6) $$\begin{array}{ll} & ∥\overset{N}{\underset{i=1}{\sum }}i(k_{i+1}-k_{i})∥<\lambda_{\text{flow}}\tau , \end{array}$$

(7) $$\begin{array}{ll} & ∥k_{t_{i}}-G_{i}{∥}_{2}<\{\begin{array}{cc} \lambda_{\text{0}}\tau & G_{i}=0,\\ \lambda_{\text{goal}}\tau & \text{otherwise}, \end{array} \end{array}$$

where the variables, *k*_*i*_ ∈ ℝ^3^, *i* = 1,…, *N* − 1, are the position in *k*-space at time *i*; *τ* controls the deviation from the constraints (1); *δ*_*T*_ is the time step; *G*_max_ and *S*_max_ are peak (3) and slew (4) constraints on the gradients; *R* is the maximum resolution used in the reconstruction (5); the sum in (6) models the phase error caused by constant flow—errors from nonconstant flow can be handled by adding norms of higher moments; *G*_*i*_ are the targets near which the trajectory should pass (7) at time *t*_*j*_, see Figure 2; and the *λ*s determine the relative priorities placed on meeting the different goals.

In the trajectories designed for this paper, the goals *G*_*j*_ are random points on the boundary sphere |*k*| = 1/*R* or at *k* = 0. Multiple traversals of *k* = 0 are not optimal for sampling, but included to facilitate calibration of gradient distortion and main-field inhomogeneity. This problem nulls the first moment at the center of the rf pulse. Higher moments, nulling at different points in the trajectory, or bounding the size of the moment with a tolerance could be encoded in the same way. The trajectories in this paper are designed to begin and end at *k* = 0, for steady-state imaging, but this is not a limitation of the method. See [2] for a detailed description of the software used to solve this optimization problem, and a discussion of variations in the design, including iterative designs in which goals are placed at low-density points for previous trajectories.

Note that the constraint on the first moment will cause an optimal trajectory to traverse parts of *k*-space not near a shortest path between the goals, *G*_*i*_, which increases the coverage of *k*-space, but there are no explicit objectives for coverage, intertrajectory spacing, or trajectory length. In [2], coverage is handled by pseudorandom assignment of goals from the vertices of a regular triangulation of the limit sphere in *k*-space.

### 2.2. Combining sets of trajectories

In this paper, coverage is assured by optimizing its effect on the point spread function (psf), which captures sampling artifacts (exactly for uniform coils, and approximately for surface coils). The ratio of the *L* ^2^ norm of the central point in the psf and the *L* ^2^ norm of the rest of the psf is maximized over subsets of a large fixed set of trajectories, each optimized as described above. Since the set of subsets of a fixed size grows factorially with the number of trajectories, only a small subset can be tested. A genetic algorithm uses information about previous subsets to test only subsets likely to have better psfs.

This approach differs from formulations, where the variables (referred to as chromosomes in genetic algorithms) are local or nonlocal control points on individual trajectories [10]. Such approaches lead to a much larger search space, and increased complexity of each search step, since the solution of the SOCP problem is the most expensive part of the optimization. In the present method, one SOCP problem is solved per trajectory (and may be solved in parallel) before application of the GA search.

Individual trajectories are 5.6 milliseconds, including 0.1 millisecond for rf excitation, and designed with gradient limits typical of a whole body clinical imager (peak gradient of 40 mTm^−1^ and a gradient slew rate 150 Tm^−1^s^−1^). For ease of comparison, we will choose enough trajectories for 1 second of sampling (178 with the above parameters).

As a first step, many trajectories are designed by randomly selecting goals on the boundary sphere (which are interleaved with fixed goals at *k* = 0). Of these, many are clearly less suitable than others. To filter out the least suitable, a small number are designed to determine an approximate threshold for the longest 20%, and the 20% closest to their own goals. Only trajectories above the thresholds are used in the initial set.

This initial large set is then partitioned into 400 sets of the desired size (168) to seed the GA. These seed sets are then evolved through 45 generations, using chromosomes consisting of integer indices into the initial set. An additional 10 trajectories were added using a “density threading” [2], whose aim is to increase sampling of voids along the *k*_*x*_ − *k*_*y*_ plane by specifically creating trajectories to pass through areas of low sampling density. Computation time refers to a PowerMac G5 2.5, running a C program calling SOCP, http://www.stanford.edu/~boyd/socp, and the Genetic Algorithm Utility Library, http://gaul.sourceforge.net.

The *objective* of the optimization is to improve the quality of the psf, as measured by Fourier transforming the (weighted) sampling density, and dividing the central value in the psf by the total energy. The method of determining the density must be the same as used in reconstructing the images, since different methods can potentially produce different artifacts even with the same trajectories [18].

To calculate the fractional sampling, both in *k*-space and in time, nearest neighbour resampling was used to calculate a sampling density, and the number of nonzero elements in the *k*-space array was computed. For example, 1 second trajectory set, 1.54% of the voxels in a 256^3^ *k*-space had nonzero values, giving one measure of under-sampling. This corresponds to 51.7% of the number of the samples that would be collected in 1 second with a receiver bandwidth of 500 kHz, giving a measure of the efficiency relative to the (unrealizable) maximum sampling rate.

### 2.3. Image reconstruction

Numerical simulations were reconstructed by simulating irregularly sampled data (500 kHz sampling) along the trajectories. Solid phantoms were approximated by cubes. Receiver coils were approximated by magnetic dipoles, with sensitivities evaluated at the center of each phantom cube. In this approximation, the signal in *k*-space corresponding to each cube is a product of sinc functions, scaled by the complex sensitivity value.

The irregular samples were then divided by the sampling density in *k*-space and resampled to a regular grid using convolution.

For multiple coils with sensitivities *S*_*i*_, the transformed images *ρ*_*i*_, were combined with two methods: weighting-by-sensitivities

(8) $$\frac{\sum_{\text{coils}}\overset{¯}{S_{i}}\cdot \rho_{i}}{\sum_{\text{coils}}\overset{¯}{S_{i}}\cdot S_{i}}$$

and conjugate gradient reconstructions [17].

A lot of analysis has been done on resampling irregular data with density variations, see [18–20] and references therein. Convolution with a fixed kernel [21] is the simplest resampling method to implement and to analyze. The optimal piecewise-linear kernel described in [22] was used. Estimating the density to use for correction was done by resampling a constant set of data points using a width-three triangular kernel. The key property of this triangular function is that it is a partition of unity for samples spaced 1, 2, or 3 grid points apart.

## 3. RESULTS

When the described algorithm was used to generate the 168-shot volume representing 1 second of imaging time, 12 hours were taken to create a working base of 4000 trajectories using randomly generated goals. An additional 13000 trajectories were rejected by the threshold test. Another 72 hours were required for the GA to select a fit subset.

**Figure 3:**
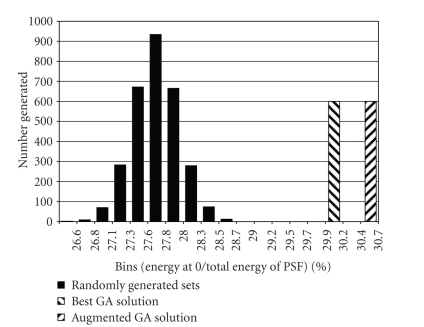
Comparison of randomly selected trajectory sets versus solutions from the GA. Note that the banded bars indicate horizontal position not counts.

Figure 3 compares the results of the GA to a histogram of 3000 randomly formed subsets. The GA set is 7.8 standard deviations better than the set, and is shown as a banded bar. Also shown is the result of adding 10 density-threaded trajectories [2].

**Figure 4:**
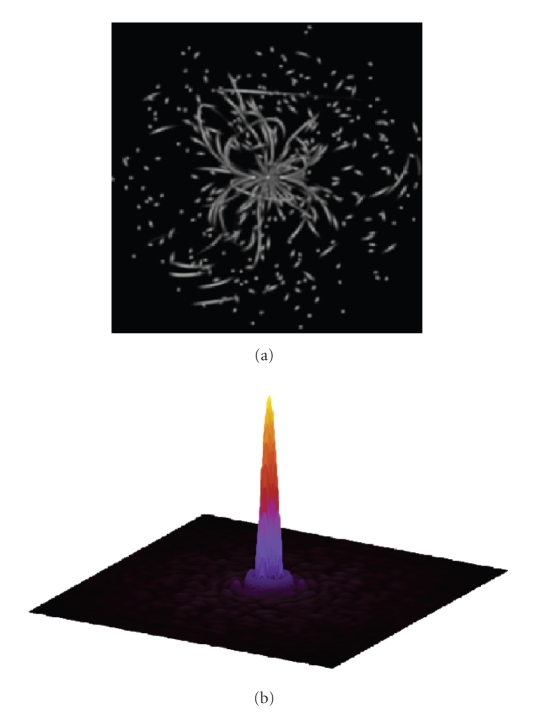
Above: cross-section of the computed sampling density used in reconstruction. (Below) cross-section of the psf, expanded using 8X Fourier interpolation.

The estimated sampling density used for density correction is shown in Figure 4. For display purposes, the corrected sampling density is shown, in which the uniformity of the bright pixels reflects the quality of the correction. Recall that only 1.54% of grid points in *k*-space contain a sample point at this reconstructed resolution.

A better measure of the quality of the set of trajectories, together with the density correction, is given by the psf (Figure 4), which predicts very little blurring and a low level of noise-like aliasing.

**Figure 5:**
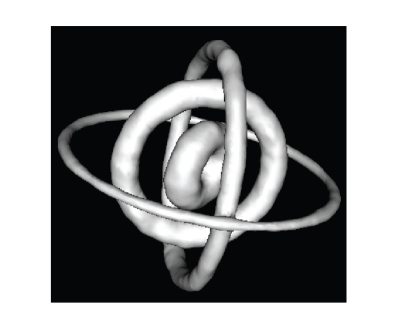
Surface rendering (OsiriX [23]) of solid rings reconstructed with 178 arcs and 32 noisy coils.

Because it is difficult to inspect more than a planar cross-section of the psf at a time, it is hard to appreciate the extent to which the noise-like aliasing accumulates in three dimensions. This is demonstrated using a numerical phantom consisting of four rings of varying sizes, Figure 5.

**Figure 6:**
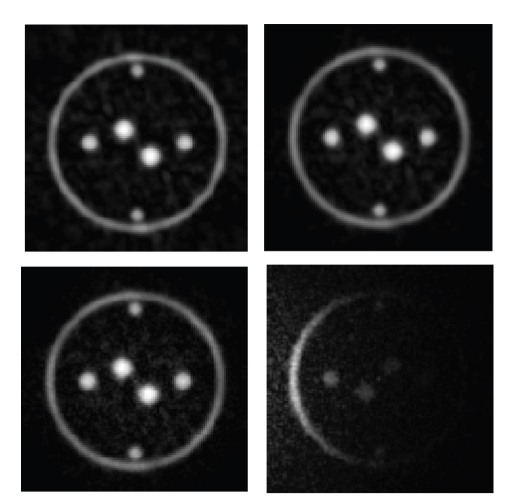
Four identical cross-sections showing, reconstruction with a uniform coil (top left), with 32 dipole coils (top right), with one dipole coil including noise (bottom right), and 32 dipole coils including noise (bottom left).

The phantom was chosen to be readily identifiable in cross-section, and in volume/surface rendering. The apparent noise will increase as a function of the total signaling volume; the phantom represents a midway point between contrast-enhanced MR angiography (with a small blood volume producing signal) and anatomical imaging. Four different reconstructions of the central *x*-*y* cross-section are shown in Figure 6.

The uniform reconstruction shows significantly more alias noise in the periphery of the image than the 32 surface-coil reconstruction. For measurement noise, this is as expected, since the coils are more sensitive near the periphery. For the noise-like aliasing artifact, a similar phenomenon is in operation. The true image is multiplied by the coil sensitivity, which is then corrected when performing multicoil combination. The artifacts are also modulated by the sensitivity, but since they are delocalized (by definition), they are not corrected during combination, and hence partially cancel each other out. This is more apparent where the sensitivities vary (the periphery) than where they are relatively uniform (the center).

The noisy multiple and single surface coil images (lower row) show typical loss of sensitivity by the center of the phantom for a single channel, which is reduced in the combined image. The 32-coil noisy image shows visually equal noise levels to the first two images, but with different (higher) frequency composition.

**Figure 7:**
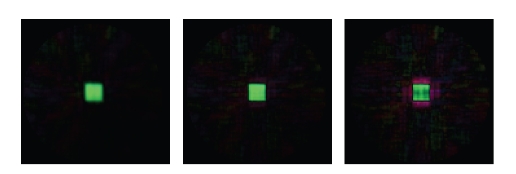
Reconstruction of a cubic phantom using 178 trajectories. (Left to right) Original 32-channel reconstruction, image showing some edge enhancement after one gradient step, and the gradient (not to scale).

**Figure 8:**
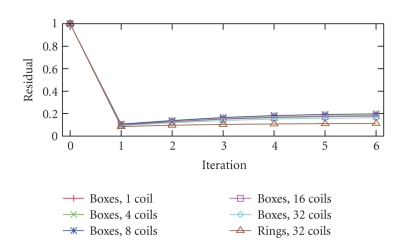
*L* ^2^ difference between true image and reconstruction after conjugate gradient iterations.

Iterative methods have been successfully used in planar imaging to reduce spiral artifacts by using a priori information about multiple coil sensitivities, notably using the conjugate gradient method [17]. This does not produce visible reductions in aliasing in this case. What it can do is reducing the effect of blurring, as is visible in the reconstruction of a 16^3^ voxel uniform box. Figure 7 shows the result of the first step common to steepest-descent and the conjugate gradient method. Figure 8 shows the residual decreasing for the first step, and starting to diverge thereafter, with two coil configurations, and simple and complex phantoms. This is not very surprising. Adapted methods have been proposed for ill-posedness [24] and round-off errors [25], but are beyond the scope of this paper.

A common cross-section is displayed of the original solution, the result after one step, and a scaled version of the gradient (using, in the electronic edition, different colors for positive and negative pixel values). What aliasing artifact is present is either unchanged or enhanced by the gradient step, while softening of the edges is clearly reduced.

## 4. DISCUSSION

The psf and numerical phantoms presented here show that Durga is a very promising approach to designing trajectories for volumetric imaging. Pseudorandom trajectory design visibly eliminates coherent aliasing artifacts in numerical simulations.

Using the GA increases the flexibility of this method and increases the quality of the solutions. There is a marked difference in quality between randomly generating subsets and using the GA to improve a population. After filtering out inferior individual trajectories, randomly selecting subsets produced surprisingly little variation in the quality of the psf. Initial attempts in using genetic algorithms based on randomly chosen initial points and unfiltered trajectories were not able to beat the heuristic in [2], so success of the GA depends very much on a careful formulation, and even then, it is unlikely that the global optimum will be reached. Unfortunately, it is also considerably more expensive. Additional work should focus on improving individual trajectories before starting the GA.

The modest improvement produced by steepest descent means that the sampling patterns are so efficient that little additional information can be extracted by a parallel reconstruction taking advantage of geometric coil information, at least with the present coils. We conjecture that the randomness optimized to make the psf flat produces a large cluster of small eigenvalues in the operator being iterated in the CG step, causing the CG to begin diverging after one iteration.

We are planning modifications of the basic trajectory design to quantify the first effect, and working with partners to collect data to evaluate the second. In any case, multiple coil reconstruction using coil sensitivities does reduce the apparent noise, which is important to end users.

We will also investigate compressed sensing reconstructions [26] which require incoherent aliasing artifacts such as those presented in this work, because in such cases “randomness is too important to be left to chance” [26].

## Figures and Tables

**Table 1:** Duty-cycle comparison for three balanced *k*-space sampling patterns.

	Spiral	Teardrop	Durga
Readout (ms)	2.40	3.43	5.50
Excitation (ms)	1.20	1.47	0.10
Rewinder (ms)	1.40	—	—
TR (ms)	5.00	4.93	5.60

Duty cycle	0.48	0.78	0.98

## References

[1] Knopp T, Kunis S, Potts D (2007). A note on the iterative MRI reconstruction from nonuniform *k*-space data. *International Journal of Biomedical Imaging*.

[2] Anand CK, Curtis AT, Kumar R (2008). Durga: a heuristically-optimized data collection strategy for volumetric magnetic resonance imaging. *Engineering Optimization*.

[3] Nayak KS, Nishimura DG Randomized trajectories for reduced aliasing artifact.

[4] Ahmad R, Deng Y, Vikram DS (2007). Quasi Monte Carlo-based isotropic distribution of gradient directions for improved reconstruction quality of 3D EPR imaging. *Journal of Magnetic Resonance*.

[5] Hargreaves BA, Nishimura DG, Conolly SM (2004). Time-optimal multidimensional gradient waveform design for rapid imaging. *Magnetic Resonance in Medicine*.

[6] Oppelt A, Graumann R, Barfuss H, Fischer H, Hartl W, Shajor W (1986). Fisp—a new fast mri sequence. *Electromedica*.

[7] Anand CK, Thompson M, Wu D, Cull T Teardrop, a novel trajectory for truefisp.

[8] Anand CK, Ren T, Terlaky T Optimizing Teardrop, an MRI sampling trajectory.

[9] Nayak KS, Hargreaves BA, Hu BS, Nishimura DG, Pauly JM, Meyer CH (2005). Spiral balanced steady-state free precession cardiac imaging. *Magnetic Resonance in Medicine*.

[10] Dale BM, Lewin JS, Duerk JL (2004). Optimal design of *k*-space trajectories using a multi-objective genetic algorithm. *Magnetic Resonance in Medicine*.

[11] Vigen KK, Peters DC, Grist TM, Block WF, Mistretta CA (2000). Undersampled projection-reconstruction imaging for time-resolved contrast-enhanced imaging. *Magnetic Resonance in Medicine*.

[12] Du J, Fain SB, Korosec FR, Grist TM, Mistretta CA (2007). Time-resolved contrast-enhanced carotid imaging using undersampled projection reconstruction acquisition. *Journal of Magnetic Resonance Imaging*.

[13] Du J, Thornton FJ, Fain SB (2004). Artifact reduction in undersampled projection reconstruction MRI of the peripheral vessels using selective excitation. *Magnetic Resonance in Medicine*.

[14] Mir R, Guesalaga A, Spiniak J, Guarini M, Irarrazaval P (2004). Fast three-dimensional *k*-space trajector design using missile guidance ideas. *Magnetic Resonance in Medicine*.

[15] Spiniak J, Guesalaga A, Mir R, Guarini M, Irarrazaval P (2005). Undersampling *k*-space using fast progressive 3D trajectories. *Magnetic Resonance in Medicine*.

[16] Barger AV, Block WF, Toropov Y, Grist TM, Mistretta CA (2002). Time-resolved contrast-enhanced imaging with isotropic resolution and broad coverage using an undersampled 3D projection trajectory. *Magnetic Resonance in Medicine*.

[17] Pruessmann KP, Weiger M, Börnert P, Boesiger P (2001). Advances in sensitivity encoding with arbitrary *k*-space trajectories. *Magnetic Resonance in Medicine*.

[18] Pipe JG, Menon P (1999). Sampling density compensation in MRI: rationale and an iterative numerical solution. *Magnetic Resonance in Medicine*.

[19] Wajer F, Lethmate R, van Osch J, Graveron-Demilly D, van Ormondt D Simple formula for the accuracy of gridding.

[20] Rasche V, Proksa R, Sinkus R, Börnert P, Eggers H (1999). Resampling of data between arbitrary grids using convolution interpolation. *IEEE Transactions on Medical Imaging*.

[21] Jackson JI, Meyer CH, Nishimura DG, Macovski A (1991). Selection of a convolution function for Fourier inversion using gridding. *IEEE Transactions on Medical Imaging*.

[22] Anand CK, Terlaky T, Wang B (2004). Rapid, embeddable design method for spiral magnetic resonance image reconstruction resampling kernels. *Optimization and Engineering*.

[23] Rosset A, Spadola L, Ratib O (2004). OsiriX: an open-source software for navigating in multidimensional DICOM images. *Journal of Digital Imaging*.

[24] Eicke B, Louis AK, Plato R (1990). The instability of some gradient methods for ill-posed problems. *Numerische Mathematik*.

[25] Strakoš Z, Tichý P (2002). On error estimation in the conjugate gradient method and why it works in finite precision computations. *Electronic Transactions on Numerical Analysis*.

[26] Lustig M, Donoho D, Pauly JM (2007). Sparse MRI: the application of compressed sensing for rapid MR imaging. *Magnetic Resonance in Medicine*.

